# When curiosity gaps backfire: effects of headline concreteness on information selection decisions

**DOI:** 10.1038/s41598-024-81575-9

**Published:** 2025-01-06

**Authors:** Marianne Aubin Le Quéré, J. Nathan Matias

**Affiliations:** 1 Cornell Tech, New York City, NY 10044 USA; 2 Cornell University, Ithaca, NY 14850 USA

**Keywords:** Human behaviour, Psychology

## Abstract

**Abstract:**

The selection of information by individuals is a basic process in democratic institutions, including journalism. Publishers attempt to attract readers with “curiosity gap” headlines that offer vague descriptions rather than summarize an article. Lab and field experiments that compare the influence of these two styles have found conflicting results on their efficacy. In this registered report, we propose a theory, based on the psychology of curiosity, to harmonize these results. We introduce and validate an automated linear scale of headline concreteness to differentiate summary and curiosity gap headlines. In a meta-analysis of 8977 headline experiments, we confirm that the effects of headline concreteness on clickthrough rates vary with the overall concreteness of other headlines. When the baseline headline is too vague, higher headline concreteness increases clickthrough rates. When headlines are too concrete, higher headline concreteness decreases clickthrough rates. These findings suggest a curvilinear relationship between information selection decisions and the amount of information conveyed in text, implying that headlines that convey just the right amount of information maximize clickthrough rates at scale.

**Protocol registration:**

The protocol for this study was pre-registered following in-principle acceptance at https://osf.io/fbzvw/ on September 21st, 2023.

The stage 1 protocol for this Registered Report was accepted in principle on 30/08/23. The protocol, as accepted by the journal, can be found at: 10.17605/OSF.IO/FBZVW.

## Introduction

When is the human desire to satisfy our curiosity the strongest, and when do institutions such as news publishers most evoke this desire? To communication scholars, these questions are part of scientific discussions about the role of language in human psychology and behavior^1–5^. These questions also matter to editors and activists in societies where democratic institutions depend on what people choose to know^6–10^.

Psychologists of curiosity argue that humans enjoy putting themselves into situations of uncertainty. In this view, an innate urge to satisfy curiosity leads people to read mystery stories or solve puzzles for fun^11^. Humans experience curiosity in the presence of an information gap, “an awareness of the absence of potentially useful or interesting information” that people wish to fill^12^. The relationship between people’s curiosity for new information and the amount of information offered by a stimulus is not linear. For example, if the difference between someone’s current knowledge and goal state seems impossibly large, a person might be intimidated to learn more on a topic. Inversely, if someone has already acquired substantial knowledge about a topic, they may not be curious to know more even if there is more they could learn. Scientists have confirmed this curvilinear relationship in a series of lab experiments and fMRI studies. At low levels of confidence and high levels of confidence in an unknown answer alike, people reported lower levels of curiosity. On average, people were most curious about the answer to a trivia question when their confidence in their answer was middling, between 0.45 and 0.55 on a scale from 0 to 1^13^. This inverted U relationship has been replicated in other studies with experiment designs involving activities that include guessing letters in words, answering general knowledge questions, and clicking on web advertisements^14–16^. Scholars argue that this curvilinear relationship between confidence and curiosity occurs because moderate levels of confidence indicate high reducible uncertainty, which increases linearly with curiosity^17,18^.

Inspired by psychological theories of curiosity, news publishers have developed a “curiosity gap” style of article headline to influence audiences to read an article^7,8^. While scientists might infer from the term “curiosity gap” that publishers are writing with knowledge of reader interests, this term of art refers solely to the genre of a headline text and not to any difference between a headline and reader knowledge. In journalism, curiosity headlines differ from more typical summary headlines, which provide a concise description of an article’s contents. Publishers reason that if information-seeking behaviors vary with the difference in individual knowledge and the information offered, then changes in the information offered should have observable effects on information seeking on average, even without access to people’s states of mind. With this theory in mind, some publishers believe that audiences will be more likely to click on headlines and read stories that pose questions, leave out information, and promise more information than they supply. Yet lab and field experiments that compare curiosity-style headlines to summary headlines have found mixed, opposing results. While some studies have found that audiences are more likely to choose summary headlines^3,19^, others have found the opposite effect^20^ or did not observe any effect^4,16^. Across nine reported lab and field experiments, three had a negative, statistically-significant effect, two had a positive, statistically-significant effect, and four were not able to observe outcomes different from random chance (Table 1).

**Table 1:** Results of published lab and field experiments testing the effect of curiosity gap style headlines compared to summary headlines. In this table, ”stimulus” refers to the style of headline participants were shown. “Measure” refers to the kind of outcome variable used, including intention to read (5-point likert, 9 point likert) or behavioral decisions to read an article (selection from list, clicks, and read behavior). ”Estimate” refers to the estimated effect on the outcome variable listed. Where statistical tables and point estimates are unavailable, we include the direction of the effect reported the paper.

Authors	Context	Stimulus	Measure	N	Estimate	P-value
Scacco & Muddiman 2019	Lab	Question	5-point likert	2057	− 0.03	p < 0.05
Scacco & Muddiman 2019	Lab	Forward reference	5-point likert	2057	− 0.01	p = 0.4
Janet et al. 2020	Lab	Question	Selection from list	900	− 0.03	p > = 0.05
Janet et al. 2020	Lab	Forward reference	Selection from list	900	− 0.26	p > = 0.05
Molina et al. 2021	Lab	Question	Read behavior	150	Negative	p = 0.003
Molina et al. 2021	Lab	Overall clickbait	Read behavior	150	Negative	p = 0.001
Lai & Audun 2014	Field	Question	Clicks	Unreported	Positive	p < 0.001
Lai & Audun 2014	Field	Question	Clicks	Unreported	Positive and negative	NA
Menon & Dilip 2002	Lab	Low information	9-point likert	131	Positive	p < 0.05

Why would scientists find so much variation in results of studies that test publisher theories about headline styles? Many experiment designs encode textual curiosity gaps as a binary, with researchers labeling a headline as curiosity-style or not. In contrast, scientific theories about psychological information gaps describe these gaps as a continuous measure of the difference in how much a person knows and how much information is offered by a stimulus^11^. Along this continuous measure, studies of curiosity have found that effect sizes vary with the magnitude of information gaps. People will not seek information if the difference between their current knowledge and the promised knowledge is too small or too great. In this literature, changes in the promised knowledge could cause a negative or a positive effect depending on the state of a person’s knowledge^13^. Since continuous differences in a person’s knowledge can result in effects with different signs and magnitude, it is possible that variations in the information provided by headlines can also result in effects with different signs and magnitudes. If so, experiments that classify headlines with binary measures could be expected to yield conflicting results, as we see in the literature.

In this paper, we test the hypothesis that conflicting results in the experimental record could be explained by the presence of a curvilinear relationship between the information offered in headlines and the decision to choose an article to read. This relationship might be observable by scientists even in the absence of information about individual knowledge or beliefs. Given central tendencies in knowledge of current affairs in the U.S.^21^, we expect that variations in the information offered by headlines would be associated with variation in curiosity on average, and consequently in decisions to read an article.

To observe the relationship between reader decisions and the information offered by a headline, we need a continuous measure of the offered information rather than the binary measure used in prior studies. Toward this end, we develop and validate a continuous, computational measure of headline concreteness, building on empirical studies of human language perception^22^. This measure estimates how well a person can imagine the concrete situation described in a given headline. Headline concreteness, a continuous measure validated by human raters, provides more precision and theoretical clarity than a binary genre of “curiosity gap” style headlines.

Comparing effects at different points of the concreteness scale requires a population of many headline experiments. In this study, we analyze a corpus of 27,616 field experiments with headlines carried out by a U.S. media publisher^23^. By coding, filtering, and meta-analyzing these field experiments, we can bridge between theory and practice with strong external validity. Based on psychological theories of human curiosity, we expect that the magnitude and direction of an individual headline’s effect on information selection will vary with the linguistic concreteness of the other headlines in the experiment.

H1: The effect on reader information selection from increased headline concreteness is negatively associated with the average concreteness of all headlines in a given A/B test.

We also test the hypothesis that greater concreteness has a positive effect on information selection behaviors in low-information experiments but a negative effect in high-information experiments. These hypotheses jointly suggest the presence of a curvilinear relationship between increasing headline concreteness and reader clickthrough rates.

H2: At the lowest observed level of average test concreteness, the effect of greater concreteness on reader information selection is positive.

H3: At the highest observed level of average test concreteness, the effect of greater concreteness on reader information selection is negative.

If these hypotheses are upheld by the evidence, then this study will be able to explain conflicting evidence in the literature and contribute to pragmatic knowledge by revealing that increasing linguistic concreteness can increase reader engagement only up to a point, at which point it causes the opposite effect. If our hypotheses are disconfirmed by the evidence, with a decrease in concreteness consistently associated with positive, negative, or null results, this study will bring clarity on the average effects of an intervention with as-yet conflicting experimental findings.

Overall, this registered report develops and proposes a way to validate a simple theoretical account that could explain competing results in the experimental record on the influence of headline styles from fields across communication, computer science, journalism, social psychology, and marketing. Furthermore, by validating a measure of headline concreteness in the field, this study also provides empirical evidence and methods that could help research in the lab explore more detailed accounts of curiosity and information selection using conditions that are closer to the field^24^.

## Methods

### The Upworthy Research Archive

Our main dataset is the Upworthy Research Archive, a set of all headline experiments run by the publisher Upworthy between January 2013 and April 2015^25^. Upworthy, a digital publisher that in 2014 was reaching about 50 million people each month, is a company dedicated to sharing “stuff that matters.” Many of Upworthy’s posts were dedicated to raising awareness of meaningful social issues and the company was largely known for their headlines seemingly geared to maximize clicks that many described as “clickbait.” For example, one of the first successes the founders saw with this tactic was to change the caption of a video from “Zach Wahls Speaks About Family” to “Two Lesbians Had a Baby and This is What They Got,” attracting 17 million views^7^. In the period covered by the archive, Upworthy received the most monthly visitors and conducted over 27,000 A/B tests. During that time, Upworthy and other publishers widely adopted curiosity gap style headlines until Facebook adjusted its algorithm to reduce how many people saw curiosity headlines or clickbait on their newsfeed^26^. Each experiment in the archive (which we refer to as a test) consists of usually 4–5 attempts to craft the perfect ‘package’ that might go viral on social media. A package is the combination of a headline and an image that act as a preview to an article. In each experiment, Upworthy used a sequential random number generator to randomly assign viewers to see different packages to test which would receive the most clicks. Randomization and data collection were conducted by software that was blind to the intent of the Upworthy staff. For each package in a test, the dataset contains how many people saw it (also known as impressions) and how many people clicked on it, allowing us to estimate the average treatment effects. In our pilot dataset, the average clickthrough rate on all tested headlines was 1.33% and the median was 1.04%.

Participants were enrolled in these randomized trials if they viewed the Upworthy website. On every article page, viewers were shown a sidebar of headline-image packages to select next. Every sidebar display included a single experiment that randomly assigned one of the available packages for a given article. Each package within a test was only tested in the same position on the page. Several years later, we retrieved the results of all experiments conducted in this period, interviewed staff, validated the data, and released it for academic use^25^.

### Concreteness measure

Scientists researching curiosity gap headlines have tended to study them using binary and categorical measures^2,27–29^. While categories of linguistic techniques provide useful guides for headline authors, categorical classifications of headlines cannot be used to investigate questions of degree. To study characteristics on a continuous scale, linguists have developed continuous measures for how much information is conveyed by language. In linguistics and embodied cognition, the concept of concreteness refers to the “degree to which the concept denoted by a word refers to a perceptible entity”^22^. Since concreteness is a measure of human perception of words, linguists have constructed and validated these continuous measures of word concreteness in experimental settings with human raters.

To create a psychologically-validated scale, Brysbaert, Warriner, and Kuperman collected concreteness ratings for forty-thousand English words and bi-grams on a scale from 0 to 5^22^. On their scale for example, the word “tomato” has a concreteness of 5 (high concreteness), the word “sound” has a concreteness of 3.7 (medium concreteness), and the word “idea” has a concreteness of 1.62 (low concreteness). Using this dictionary in combination with algorithms for identifying proper nouns, we propose a method for researchers to compute a continuous measure from 0 to 5 for the average concreteness of a headline.

**Fig. 1:**
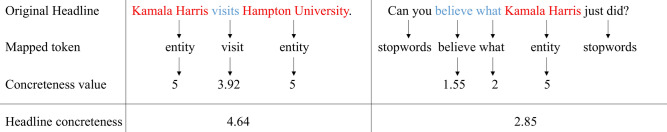
Example process for assigning a concreteness score for each headline. Tokens in red (e.g. “Kamala Harris”) map to entities, which always receive a score of 5 or maximum concreteness. Tokens in blue are words that are assigned a concreteness score according to concreteness ratings developed by Brysbaert, Warriner, and Kuperman^22^. Tokens in black are common stopwords, which are excluded from the analysis. Where applicable, tokens are mapped to their root word (e.g. “visits” → “visit”) before being assigned a concreteness score. The overall headline concreteness score is then calculated by taking the average of all word and entity concreteness ratings (e.g. (5 + 3.92 + 5)/3 = 4.64).

In this study, we calculate an average concreteness score for each headline in the Upworthy Archive by applying linguistic measures of concreteness to all words in a headline and calculating the mean^22^. We first identify any person, place, or organizational entities in a headline using the spaCy package Named Entity Recognition tagger^30^, and encode these entities with the highest concreteness score of 5. We then split our headline into a list of tokens and remove standardized English stopwords^31^ from the headline, with exception of forward-reference words (e.g. “this,” “these”) and pronouns (e.g. “she”, “they”) that journalism scholars argue are used in headlines to heighten the information gap^2,27^. We ignore punctuation and cardinal numbers. From the remaining list of tokens, we take an iterative approach to mapping each token to its concreteness rating, checking between each step if the words maps to a concreteness rating. At each step, if we cannot yet retrieve a concreteness rating for a token, we first attempt to retrieve a singular version of the token (e.g. “elephants” → “elephant”), a present tense version (e.g. “lounged” → “lounge”), or a base adjective (e.g. “greatest” → “great”). If these steps all fail and a word is hyphenated, we take the average of both words (e.g. “super-spectacular” → “super”, “spectacular”). If the headline includes at least one word with three or more letters that cannot be tagged with a concreteness score using this method, we exclude the headline from our analysis. These words are frequently slang, recent terms, terms that Upworthy has coined, and words that are not recognised by the natural entity recognition software, for example “Obamacare” or “Wond-tacular.” Using this technique, a majority of headlines can be tokenized, tagged with average concreteness scores, and retained for further analysis. Although we take a number of steps to match words to their concreteness ratings, we eliminate approximately 17.1% of total headlines from our sample which contain a long word that we cannot match. Figure 1 demonstrates our method with one high concreteness headline and one low concreteness headline.

#### Concreteness measure validation

Because our computational measure of headline concreteness extends prior work on measuring language concreteness, we have validated the new measure. Unlike other studies, this research measures the concreteness of headlines rather than individual words. Other researchers have correlated the single-word average concreteness ratings collected by Brysbaert, Warriner, and Kuperman^22^ with ratings assigned to multiword expressions. They found a correlation of *r* = 0.67 between average human ratings of whole expressions and the mean ratings of the individual words within those expressions^32^. They also found that the reliability of these average concreteness ratings decreased for expressions with more words. Our study applies these methods to full headlines, which are more complex than multiword expressions. We also also used Named Entity Recognition to rate words about people, places, and organizations with high concreteness. Given these differences, we conducted a validation study to compare the computationally-generated headline concreteness measurement with human judgments of a headline’s concreteness.

In our validation study, participants (*N* = 176) rated headlines from 1 to 5 on a scale of most abstract to most concrete. We then evaluated the computationally-generated concreteness ratings by comparing them to these human judgments with intraclass correlation and Pearson correlation. We assigned each headline to receive 10 ratings at minimum, following the approach taken in previous studies^22,32^. Procedures were approved by the Cornell Institutional Review Board, performed in accordance with relevant guidelines and regulations, and we collected informed consent from all participants. More information about participant demographics is included in the Supplementary Materials.

We closely modeled the participant experience on the design of previous studies of linguistic concreteness. First, we trained participants to recognize headlines that were concrete, abstract, and in-between concrete and abstract, with three headline examples from the Upworthy Archive. We used the training materials developed by Brysbaert et al.^22^ for their word concreteness labeling study, replacing any mention of “word” with “headline”. Participants were told to rate headlines from 1 (most abstract) to 5 (most concrete). They could also indicate if they did not understand a headline. Participants next completed a set of calibration ratings which consisted of eight headlines selected by the researchers to represent the full scale of abstract to concrete. Finally, each participant was shown a random subset of 20 headlines from a total sample of 320 headlines that were validated. Participants were also exposed to one attention check. We excluded 13 unique participants in total: those whose responses on the calibration ratings correlated less than *r* = 0.2 with the mean of all other responses (10 participants), those whose ratings had zero variation (1 participant), those who failed the attention check (1 participant), and those who revoked their consent after completing the study (1 participant). The final validation study included 176 participants.

The 320 headlines in our validation study were drawn from the Upworthy Archive using a random stratified sample. To include the full range of concreteness ratings, we drew 80 headlines from four ranges of computationally-assigned concreteness: 0–2.5, 2.5–3, 3–3.5, and 3.5–5. We based this sample size on a power analysis for a minimum correlation of *r* = 0.2 at 0.95 power. Since there is a known correlation between the number of words in an expression and its perceived concreteness, we sampled headlines that all had between 14 and 16 words (45% of all headlines fell within this range).

Overall, we collected 3565 valid ratings for 320 headlines, with an average of 11 ratings per headline. To evaluate the reliability of the computational measure of concreteness, we estimated the intraclass correlation coefficients between human judgments and the computational measure. We calculate the first ICC, which expresses how much of the variability was due to systematic rater differences, and the second ICC, which captures the overall reliability of the average rating. The first and second ICCs are 0.26 and 0.79 respectively, indicating good overall reliability of human ratings. The Pearson correlation between the mean of a headline’s participant ratings and our computational concreteness metric is *r* = 0.61 (*p* < 0.0001). Based on this result, we conclude that our automated concreteness measure is a viable proxy for human perception of the concreteness of a headline.

### Meta-analysis

Our data follows a hierarchical structure, so to test our hypotheses we fit a multilevel model with an interaction between individual-level headline concreteness and group-level A/B test concreteness^33^. The coefficient, significance, and sign of the interaction term informs H1. To test H2 and H3, we then perform two simple slopes tests to understand the sign and significance of the slope at the minimum and maximum value of mean test concreteness.

**Fig. 2:**
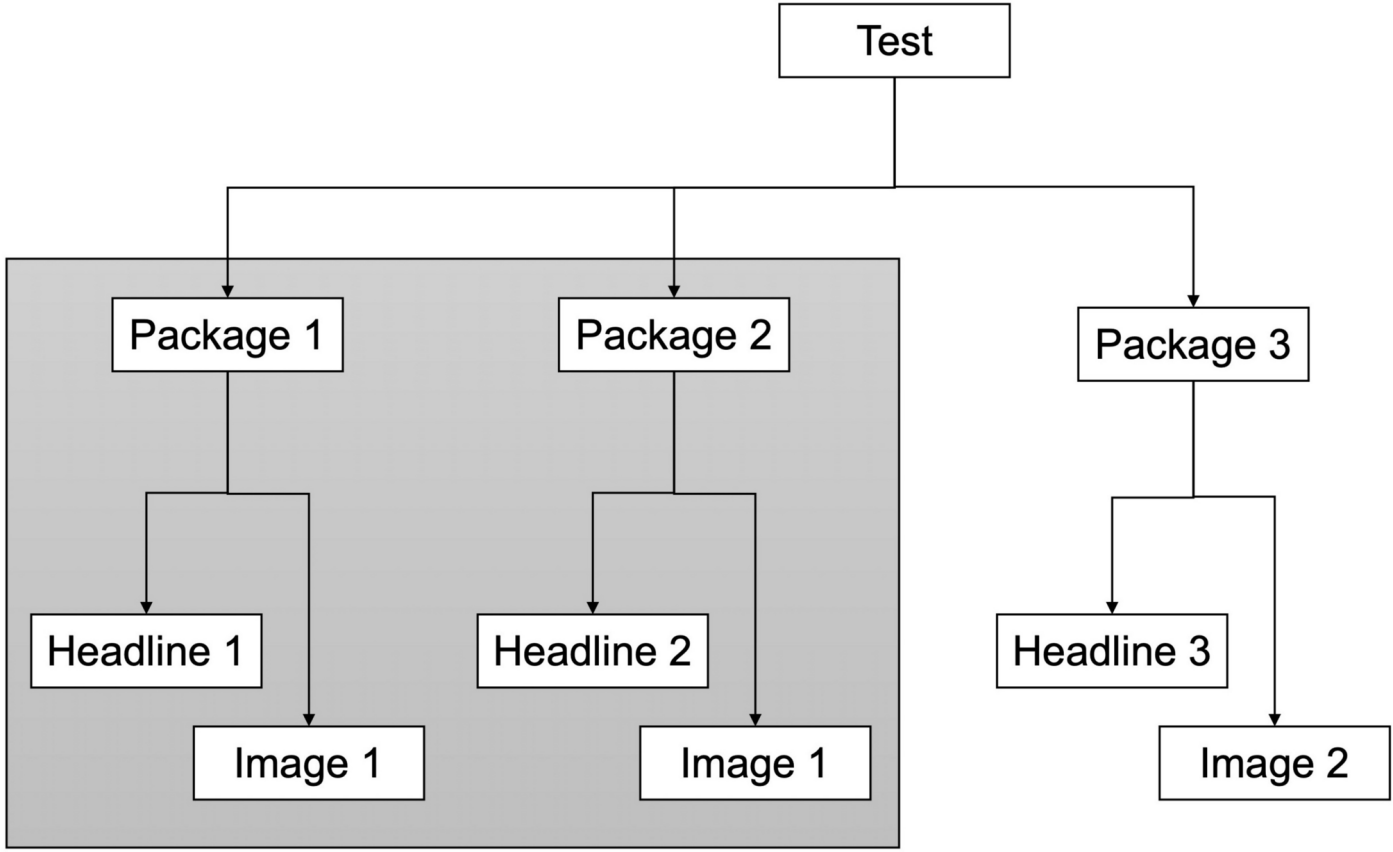
Illustration of our setup for deciding which headlines within a single A/B Test can be compared. Even though all headlines are distinct, they cannot all be validly compared, as the image is different. In this example, Headline 1 can be compared with Headline 2, as the image is constant. However, Headlines 1 and 2 cannot be validly compared with Headline 3, as the image is different. In this example, we would exclude Headline 3 from our analysis.

Tests conducted by Upworthy sometimes varied the image displayed alongside a headline, so we followed guidance to split these tests into bundles of packages that use the same image^25^. Since packages within a test were randomly assigned, we can create these subsets of packages with confidence that each participant had an equal chance of being assigned to a given headline. Two headlines are comparable if they are a part of the same test, and only if the accompanying image shown to participants as a stimulus is the same. If the image is different, the headlines cannot be validly compared since we cannot determine whether any potential effect would be confounded by image differences. If there are no two headlines in a test that can be compared, we exclude the test from our analysis. In some cases, we also have multiple sets of headlines, where not all headlines can be compared with each other. In those cases, we split each set of comparable headlines into its own test. We thus have two levels for the multi-level analysis: individual-level headlines are nested within a group-level A/B test. Figure 2 illustrates this selection and nesting process.

We construct a binomial random-intercepts, random-slopes multilevel model using headline-level observations. The dependent variable we measure is the clickthrough percent on a package, defined as *number of total impressions/number of clicks*. We opt for a binomial model since this best represents our data, with potential outcomes ranging from 0 (a headline was never clicked on) to 1 (a headline was clicked on by every user who saw it). We include three main independent variables in our model. The first is the *headline concreteness*, which is the concreteness assigned by our algorithm to an individual-level headline. This variable is centered at the group mean and divided by two standard deviations. The second independent variable is the *test concreteness mean*, which is the group-level mean concreteness of all of the headlines in a test. The final independent variable is the interaction between *headline concreteness* and *test concreteness mean*, which captures how the effect of concreteness on clickthrough rate is moderated by the mean of all headlines in a test. Our model setup resembles that of other scholars who have analyzed the Upworthy Archive^34,35^.

Our final multilevel model, with *i* representing the individual-level headlines and *j* representing the group-level tests, is a binomial random-intercept, random-slopes model, defined as the equation:

$$\begin{aligned} {\text{logit}}({\text{Y}}_{{{\text{ij}}}} ) & = (\beta_{0} + \upsilon_{{0{\text{j}}}} ) + (\beta_{{1}} + \upsilon_{{{\text{1j}}}} ){\text{headline concreteness}}_{{{\text{ij}}}} + \beta_{{2}} {\text{test concreteness mean}}_{{\text{j}}} \\ & \;\;\; + \beta_{{3}} {\text{headline concreteness}}_{{{\text{ij}}}} \times {\text{test concreteness mean}}_{{\text{j}}} + \varepsilon_{{{\text{ij}}}} \\ \end{aligned}$$

To test H1, we assess the interaction term between headline concreteness and test concreteness mean. This interaction term tests whether the magnitude of concreteness effect on clickthrough rate varies negatively based on a test’s overall average headline concreteness (H1). The decision rule for this test is *p* < 0.05.

To test the hypothesis that a relationship between effect sizes and mean concreteness explains why prior experiments have observed both positive and negative effects (H2 and H3), we perform two simple slopes tests to assess the significance of the slope of our model at different values for mean test concreteness. The decision rule for this test is *p* < 0.05. We anticipate that, within the space of possible values for mean test concreteness, there is an area at low mean test concreteness where the positive slope (headline concreteness is significantly likely to positively vary with clickthrough rates) is significant and an area at high mean test concreteness where the negative slope (headline concreteness is significantly likely to negatively vary with clickthrough rate) is significant. In a simulated power analysis from pilot data, we estimated that this study will need at least 3,500 experiments for 99% power across all hypotheses. We use the *lme4* R package^36^ to construct our model, and the *reghelper* R package^37^ to run the simple slopes test.

Prior to the final confirmatory analysis of this dataset, we conducted a pilot analysis, using the time-stratified exploratory dataset of 4837 experiments provided by the archive creators^25^. In our pilot analysis, we applied the inclusion and exclusion steps described in the “Methods” section, which resulted in a total 2335 tests to be meta-analyzed. Across the exploratory dataset, we found a significant negative interaction between headline concreteness and mean test concreteness (*p* < 0*.*0012) (H1). In our pilot dataset, the simple slopes analysis found that the effect of headline concreteness on clickthrough rates is positive (*p* < 0.0012) at the lowest occurring value of mean test concreteness (H2), and negative (*p* < 0.0012) at the highest occurring value of mean test concreteness (H3). The full pilot analysis is detailed in the Supplementary Materials.

## Results

We apply the headline concreteness metric to the 17,867 tests and 85,830 headlines that are part of the confirmatory data set. 70,546 of the pilot headlines can be tagged using our headline concreteness metric, so we continue the analysis with 82.2% of packages included.

We then sub-select valid tests for comparison. The next requirement to satisfy is that, within each test, the accompanying image is constant. If multiple images were tested, we break up the tests as described in Fig. 2. This process yields a total of 49,806 one-image tests where the image is held constant. Many of these compound tests have only one tested headline. A test from this wider set can only be selected for comparison if there are at least two different tested headlines. From this wider set, we identify 8977 one-image tests that meet our definition of a valid test, which include 35,910 non-unique headlines (31,077 unique headlines) tagged with concreteness.

As a final step before running our analyses, we standardize the headline concreteness variable. In this process, we subtract the headline concreteness from the sample average test concreteness, then divide by two standard deviations. We validate that the resulting headline concreteness and average test concreteness are not correlated, *r*(35,907) <  − 0.0005*, p* = 0.999.

We first test H1 by running a binomial, random-intercepts and random-slopes multilevel model with an interaction between individual-level *headline concreteness* and group-level *test concreteness average*. We summarize the results of the model in Table 2. This model was performed in *R* with the *lme4* package. We constructed the binomial model using the logit link function and the *bobyqa* optimizer.

**Table 2:** Final analysis table of the multilevel model. The outcome variable is the logit of the clickthrough percentage on an article based on its headline. There is a significant negative interaction between headline concreteness and mean test concreteness, implying that the effect of headline concreteness on clickthrough rates varies negatively with mean test concreteness.

	Dependent variable:
	Logit (Clickthrough percentage)
*Fixed effects*	
Headline concreteness	0.168** (0.066, 0.271)
Mean test concreteness	0.102*** (0.051, 0.153)
Headline concreteness × mean test concreteness	− 0.058*** (− 0.091, − 0.025)
Constant	− 4.929*** (− 5.085, − 4.772)
*Random effects*	
Variance (Headline Concreteness)	0.1333 (*SD* = 0*.*3651)
Variance (Intercept)	0.4269 (*SD* = 0*.*6534)
Observations(i)	35,910
Observations(j)	8977
Log Likelihood	− 160,545.0
Akaike Inf. Crit.	321,104.0
Bayesian Inf. Crit.	321,1563.5

Note:* p < 0.05; ** p < 0.01; *** p < 0.001.

The interaction between headline concreteness and average concreteness is negative at – 0.058 (*p* < 0.001). The negative value of the interaction reflects that we expect the relationship between concreteness and clickthrough rate to be less for higher values of mean test concreteness. After performing the Holm correction for multiple hypotheses, p is adjusted to < 0.0013. Since the p-value is less than 0.05, and the coefficient is negative, we reject the null hypothesis of no relationship (H1). We conclude that the effect of concreteness on clickthrough rates varies for different levels of mean test concreteness, and that this relationship causes the effect of concreteness on clickthrough rates to be less for higher values of mean test concreteness.

**Fig. 3:**
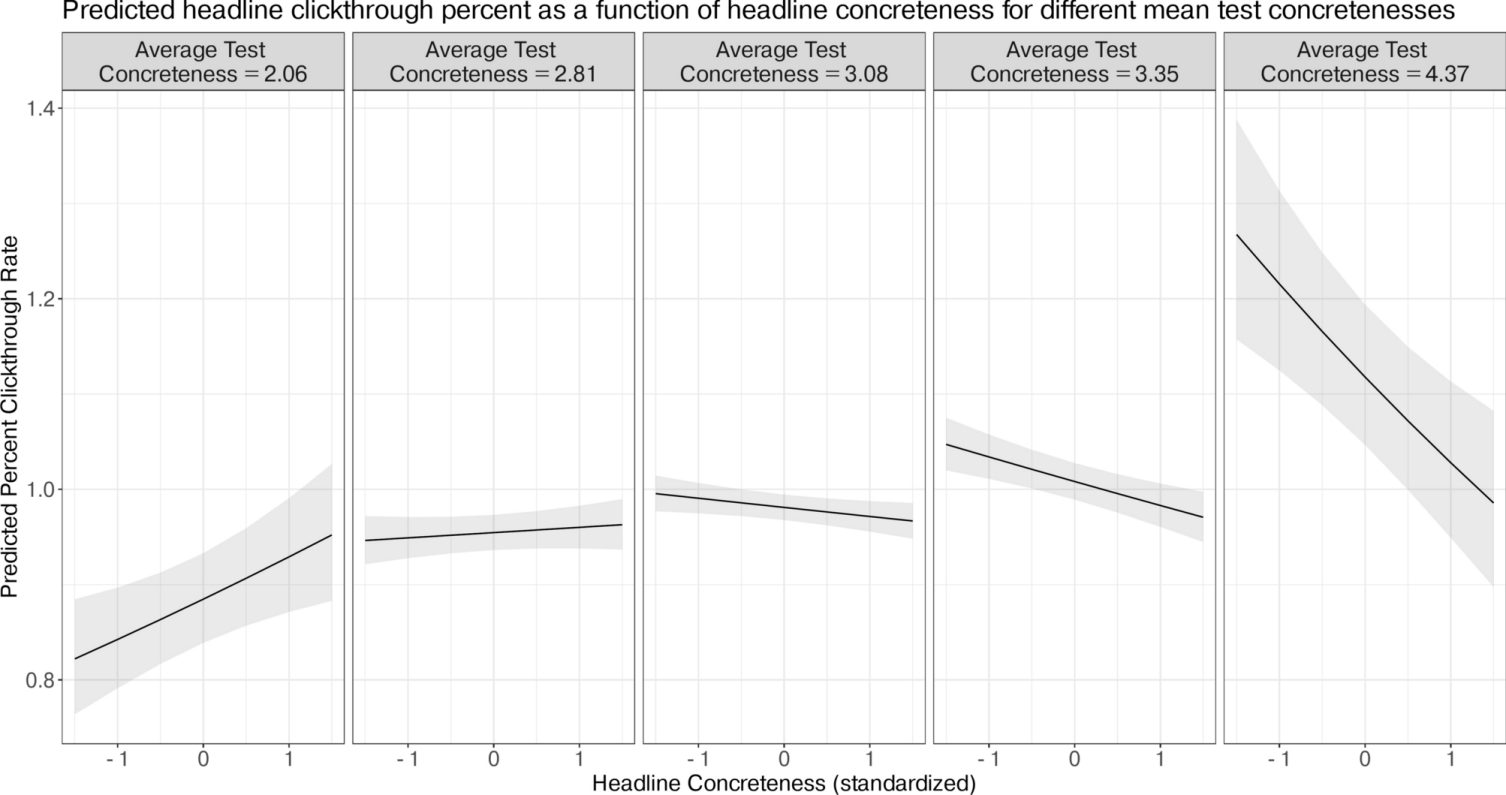
Estimated marginal means of headline concreteness on predicted percent clickthrough rate, for different levels of mean test concreteness. The levels of mean test concreteness selected are: lowest value (2.06, left), one standard deviation below the mean (2.81, second from left), mean (3.08, middle), one standard deviation above the mean (3.35, second from right), highest value (4.37, right). The headline concreteness is depicted from − 1 (2 standard deviations below the mean) to 1 (2 standard deviations above the mean). Overall, we observe an upside-down U-shape, where clickthrough rates appear to vary positively with headline concreteness when average test concreteness is lower, vary negatively with headline concreteness when average test concreteness is higher, and not significantly vary when average test concreteness is in the middle.

**Fig. 4:**
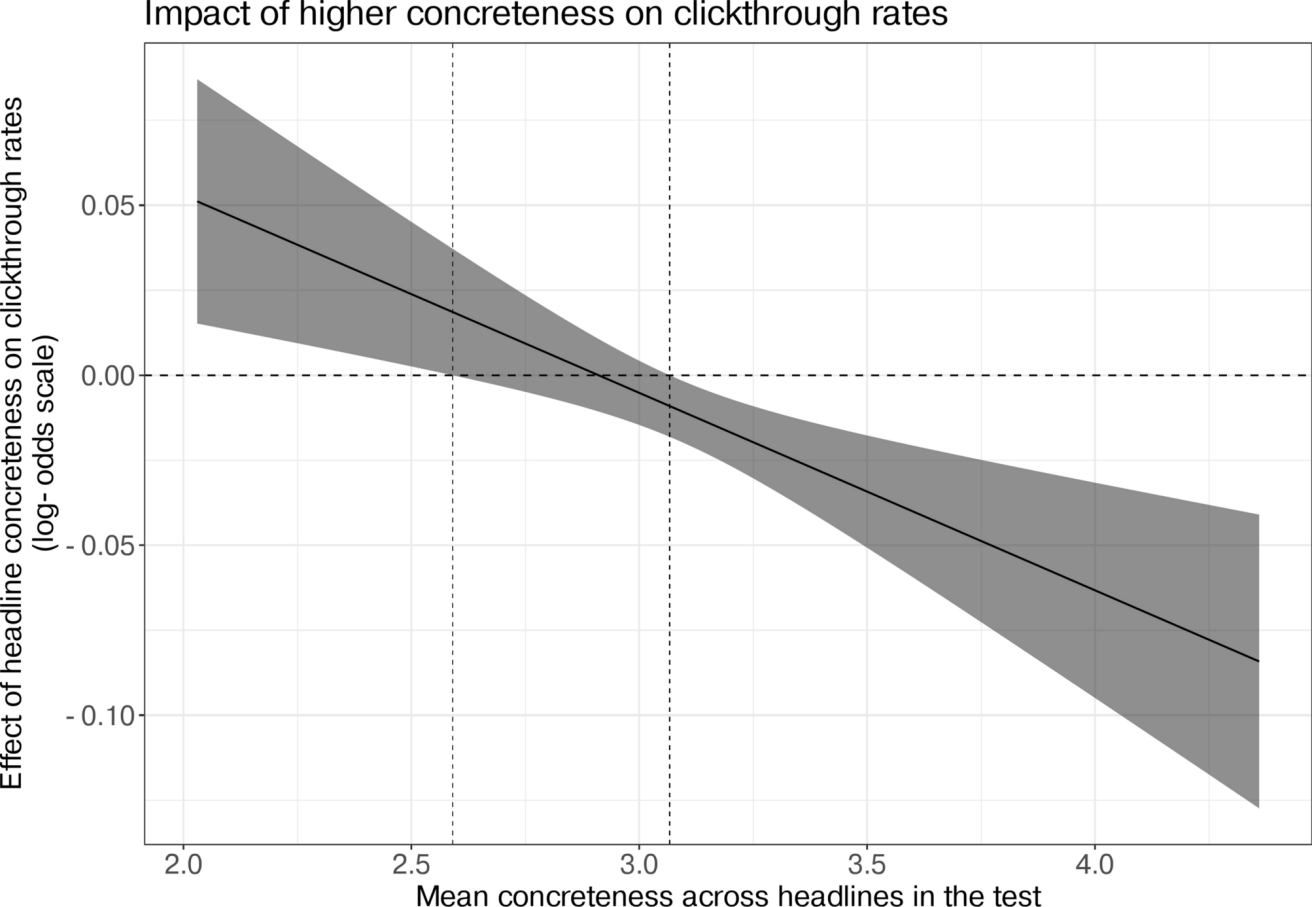
Johnson-Neyman plot based on Upworthy data: figure shows the estimated effect (y-values) of headline concreteness on the clickthrough percent as a function of mean test concreteness (x-values). Because the confidence intervals cross zero (marked by vertical lines at 2.58 and 3.06), the figure implies that more concrete headlines correlate significantly with higher clickthrough rates at lower levels of concreteness and lower clickthrough rates at higher levels of concreteness.

We also test the second and third hypotheses that the relationship between mean concreteness and headline concreteness might explain why prior experiments have observed both positive and negative effects. As a first step, we visualize the conditional effects of the interaction for different values of mean test concreteness. Figure 3 illustrates that the slope is positive for lower values of mean test concreteness and negative for higher values of mean test concreteness. This figure shows that clickthrough rates vary positively with headline concreteness when all headlines in a test are low, and clickthrough rates vary negatively with headline concreteness when all headlines in a test are high. To confirm this intuition statistically, we perform simple slopes tests, which allow us to calculate the expected slope and its confidence interval for the interaction between headline concreteness and mean test concreteness at specific values of headline concreteness. We use the *reghelper* R package to test the simple slopes, and an online calculator (http://www.quantpsy.org/interact/hlm2.htm) to calculate the bounds of mean test concreteness for which the slope is significant and visualize the analysis results. At the lower bound, the slope of the interaction is positive and significant for mean test concreteness values less than 2.58 (8.7% of tests in the confirmatory dataset), and the slope is negative and significant for mean test concreteness values greater than 3.06 (50.9% of tests in the confirmatory dataset). Figure 4 visualizes these bounds using a Johnson-Neyman plot. If an A/B test consists of two headlines that differ by one unit of standardized concreteness and which collectively have a low mean concreteness of 2.06, our model estimates that the clickthrough rate of the higher-concreteness headline will be 0.05 higher (*holm* − *adjusted p* < 0.007) on the log-odds scale than the low-concreteness headline, and thus be more likely to be clicked on. To give a practical example, at this lower bound, the model predicts that a higher-concreteness headline (1 standard deviation above the mean) would yield a 0.91% clickthrough rate, while a lower-concreteness headline (1 standard deviation below the mean) would yield a 0.86% clickthrough rate (5.5% decrease). Conversely, if an A/B test consists of two headlines that differ by one unit of concreteness, with a high mean concreteness of 4.41, our model estimates that the higher-concreteness headline will have a clickthrough rate that is 0.08 lower (*holm* − *adjusted p* < 0.0005) on the log odds scale than the than the low-concreteness headline, and thus be less likely to be clicked on. Practically, at this upper bound, a lower-concreteness headline (1 standard deviation above the mean) is predicted to yield a 1.17% clickthrough rate, while a higher-concreteness headline (1 standard deviation below the mean) would yield a 1.06% clickthrough rate (9.9% decrease).

## Discussion

This study explores how headline concreteness impacts information selection decisions in a field study of A/B-tested digital media headlines. In this analysis, we find that the impact of headline concreteness on clickthrough rates depends on the average concreteness of all headlines in an A/B test (H1). Practically, this finding means that changing the concreteness of a headline might not universally increase or decrease clickthrough rates. Instead, the effect on clickthrough rates of changing headline concreteness depends on how concrete the baseline headline is.

More specifically, we show that when all headlines in a test have low concreteness, higher-concreteness headlines are more likely to be clicked (H2), and conversely when all headlines in a test have high concreteness, higher-concreteness headlines are less likely to be clicked on (H3). Increased headline concreteness positively predicts clickthrough rates for low levels of concreteness, and negatively predicts clickthrough rates for high levels of concreteness. From these findings, we conclude that clickthrough rates for a headline are maximized when the headline possesses a “middling” amount of concreteness–headlines are not too vague and not too specific. We note, however, that this finding does not appear to be symmetrical. The model predicts that only 8.7% of headlines published by Upworthy (*concreteness* < 2.58) benefit from becoming more concrete, leading to significantly higher clickthrough rates, versus predicting that for 50.9% of headlines (*concreteness* > 3.06), increasing concreteness significantly decreases clickthrough rates. For headlines that have a “middling” level of concreteness (*concreteness* >  = 2.58 *and concreteness* <  = 3.06), manipulating headline concreteness is not expected to significantly impact clickthrough rates.

Our findings have theoretical implications for the fields of psychology, communication, and computer science. To psychologists, our findings tie into prior information gap theories that suggest that a medium difference between current knowledge and anticipated knowledge maximizes curiosity^11^. While we do not directly measure individual-level curiosity or information gaps, scholars have proposed that concreteness can be a way to measure the amount of information conveyed by text^38^. Other scholars studying information gaps have manipulated concepts that also vary the information offered in curiosity-inducing stimuli, for example, the number of letters in a missing word^14^. In this way, our findings do corroborate the idea of a curvilinear relationship between curiosity-driven information selection decisions and the amount of tangible information contained in text (or left to the imagination). Future research could further explore the link between concreteness and curiosity using the measures we have validated in this study, combined with more direct individual-level measurement of perceptions and beliefs. In general, this work formalizes and validates concepts related to the information gap theory of information selection. In the communication discipline, our findings showcase an alternate approach to classifying headlines with a binary “summary headline” or “clickbait headline” definition. Our findings also help explain conflicts in prior experimental findings where clickbait headline features are sometimes clicked on more^16,39^, and sometimes less^3,4,19^. In further work, communication scholars may also wish to understand the relationship between concreteness, curiosity, clickthrough rates, and forms of civic engagement—for example, making a political donation or signing a petition. For computer scientists, we also suggest that more effective clickbait detection models and datasets in the future should go beyond the usual binary “clickbait” or “non-clickbait” classification^40,41^ to include continuous measures of linguistic features that are grounded and validated in human psychology. We present headline concreteness as one such validated feature that could be incorporated into these clickbait detection and generation models.

This study can also inform practitioners working to write headlines that summarize article content while also appealing broadly to audiences in an information-rich environment. While clickbait is often touted as an easy way to get more clicks, our results suggest that there is such a thing as omitting too much information. Even when practitioners strive for engagement, making a headline too vague or too concrete can result in lower engagement, something that happened often to Upworthy. To help practitioners, we provide lists in the supplemental materials of headlines from the Upworthy corpus that would be considered too vague (*concreteness* < 2.58) and too concrete (*concreteness* > 3.06).

This study also offers a methodological contribution through a validated sentence concreteness score. Prior research has yielded extensive word-level and multi-word expression dictionaries of concreteness^22,32^. We extend this methodological research to sentence-length sequences, using computational linguistic techniques that tag people, places, or organizations as high-concreteness entities in text, and show that these automated concreteness ratings align with human judgments. We have made this concreteness tagging easily available for other researchers to use through a python package, *sentence concreteness* (https://github.com/maubinle/sentence_concreteness).

This study also has several limitations. As is the case with most meta-analyses, experiments are not drawn from a representative distribution of possible stimuli. In scientific research, we expect that prior literature influences subsequent literature; the same can occur with headline writers. If headline writers were learning from past experiments, they may have prioritized stimuli that they considered most likely to be effective, leaving some kinds of stimuli under-explored. These experiments were also conducted with a specific audience. Tests were only shown to people who were on the Upworthy website, an English-language site that primarily focused on American audiences. Upworthy was known for their clickbait headlines, so it is possible that the people who would have been exposed to these headlines were those who had a propensity for this kind of content, a preference that may be an individual-level characteristic^42^. We cannot conclusively rule out the possibility that this population uniquely exhibited the non-symmetrical finding that fewer headlines are considered *too vague* than *too concrete*—the Upworthy audience may have a skewed preference for less concrete headlines.

## Conclusion

Communication scholars and media practitioners alike have turned to the psychology of curiosity to understand why people choose to read articles and how to influence them to do so. Information gap theories of curiosity have inspired both publisher practices and scientific research on the most influential styles of news headlines. In this registered report, we reviewed the prior literature, described discrepancies in the experimental record, and proposed a theory based on the psychology of curiosity that could explain this discrepancy.

With the confirmatory analysis of 8977 experiments, we validated that increased headline concreteness can both positively and negatively predict headline clickthrough rates. These findings imply that there is an “optimal” level of headline concreteness that maximizes clickthrough rates, and tie into psychological theories of the information gap.

## Supplementary Information

Supplementary Information.

## Data Availability

The data used for this study, including pilot and full data, is documented and publicly made available upon request at https://upworthy.natematias.com.

## Code availability

All code used for this analysis is made publicly available and documented at https://osf.io/fbzvw/ so that other researchers can reproduce and amend our process. Additionally, we make available a python package that allows researchers to tag sentences easily with a concreteness score at https://github.com/maubinle/sentence_concreteness.
