# Supplementary material for: When curiosity gaps backfire: effects of headline concreteness on information selection decisions
Source: Sci Rep. 2025 Jan 6;15:994. doi: 10.1038/s41598-024-81575-9 (PMC11704130; doi:10.1038/s41598-024-81575-9)
Supplement: Supplementary file 1 — Supplementary Information. [file 41598_2024_81575_MOESM1_ESM.pdf]

Supplementary Materials

When Curiosity Gaps Backfire: Effects of Headline Concreteness on Information Selection Decisions

ABSTRACT

These supplementary materials include the concreteness validation survey, the full pilot data analysis submitted at Stage 1, and the full data analysis. We include documentation of our process for managing new information about problems with randomization in 22% of the experiments in the Upworthy Archive after submitting our Stage 1 registered report. We include an analysis of the full archive inclusive of these unreliable experiments, showing that excluding or including these experiments does not materially alter the findings. We also include auxiliary materials relevant to our analysis.

1 Validation Survey Analysis

In this supplementary section, we include more details about the validation study we ran to validate our measure of concreteness.

Participants had a mean age of 35.4 ($SD = 11.1$). In our sample, 64 participants did not have a college degree, 85 participants had a post-secondary degree but no postgraduate degree, and 28 participants had a postgraduate degree. Three participants indicated that English was not their first language. On average, participants completed our study in 6 mins and 10 seconds, and each participant was paid $2 for a completed study, resulting in a $19.46 hourly rate. We used the platform Prolific to recruit participants.

We make a few notes about specific headlines. Despite our efforts to assign each headline to raters 10 or more times, there were four headlines which were only rated nine times. Additionally, due to a bug in our initial survey construction, the first 20 participants all viewed the same two additional headlines last. Since the inclusion or exclusion of these headlines do not alter the correlation or significance of our results, we opt to include all headlines in the reported analysis.

To help exemplify our results, Supplementary Figure 1 shows the overall correlation between all human-rated observations and automated concreteness ratings.

2 Stopwords

We relied largely on the standard list of NLTK stopwords for our choice of words to ignore when tagging concreteness. However, we removed from this list words that are often used by journalists for forward-reference. These additional words we chose to exclude are: [’these’, ’this’, ’it’, ’what’, ’he’, ’she’, ’her’, ’him’, ’his’, ’they’, ’them’, ’this’, ’what’, ’here’, ’how’]

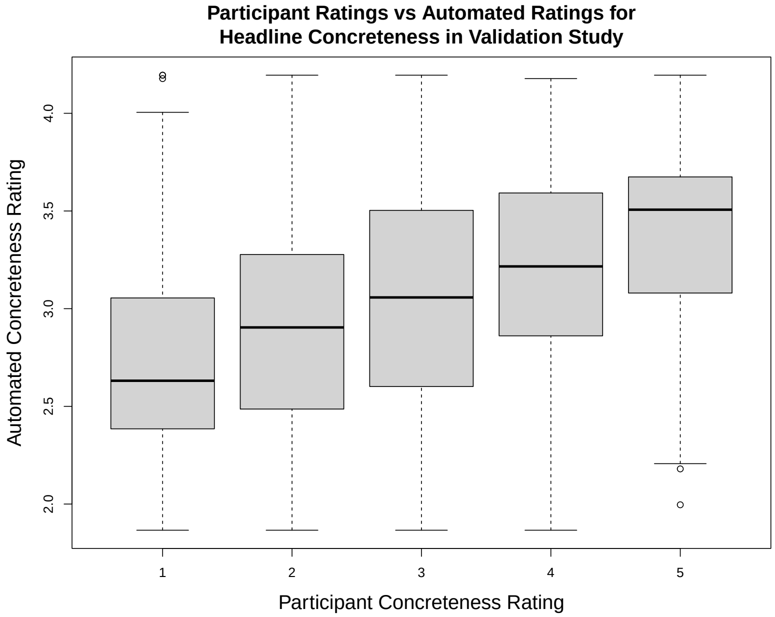

Supplementary Figure 1. Boxplot shows the alignment between individual headline participant concreteness ratings and the automated concreteness measure. The x-axis shows the participant-assigned concreteness value for a headline, and the y-axis shows the automated concreteness value for the headline. The middle line represents the median, and the box encompasses the interquartile range. There is a positive correlation between the participant-assign ratings and the automated concreteness score.

3 Pilot Data Analysis

We apply the headline concreteness metric to the 4,837 tests and 22,666 headlines that are part of the pilot data set. 18,791 of the pilot headlines can be tagged using our headline concreteness metric, so we continue the analysis with 82.9% of headlines included. The headlines that are excluded contain longer words that we cannot map to concreteness, and thus we cannot reliably tag them with an overall concreteness score. Examples of words that we cannot tag at this stage include “Obamacare,” “ebola,” and “kinda.”

We then sub-select valid tests for comparison. The first requirement to satisfy is that, within each test, the accompanying image is constant. We thus break up the tests where multiple images were tested. This process yields a total of 10,798 one-image tests where the image is held constant. Many of these compound tests have only one tested headline. A test from this wider set can only be selected for comparison if there are at least two different tested headlines. From this wider set, we identify 2,335 one-image tests that meet our definition of a valid test, which include 9,101 non-unique headlines tagged with concreteness.

As a final step before running our analyses, we standardize the headline concreteness variable. In this process, we subtract the headline concreteness from the sample average test concreteness, then divide by two standard deviations. We validate that the resulting headline concreteness and average test concreteness are not correlated, $r(9099) < -0.0004, p = 0.96$.

In the pilot analysis, we first test H1 by running a binomial, random-intercepts and random-slopes multilevel model with an interaction between individual-level *headline concreteness* and group-level *test concreteness average*. We summarize the results of the model in Table 2. This model was performed in *R* with the *lme4* package. We constructed the binomial model

using the logit link function and the *bobyqa* optimizer. Based on the pilot data, the interaction between headline concreteness and average concreteness is negative at -2.915 ($p < 0.004$). The negative value of the interaction reflects that we expect the relationship between concreteness and clickthrough rate to be less for higher values of mean test concreteness. After performing the Holm correction, p is adjusted to < 0.011. Since the p-value is less than 0.05, and the coefficient is negative, we reject the null hypothesis of no relationship in this exploratory pilot data (H1). From the pilot data, we would conclude that the effect of concreteness on clickthrough rates varies for different levels of mean test concreteness, and that this relationship causes the effect of concreteness on clickthrough rates to be less for higher values of mean test concreteness.

Supplementary Table 1. Example analysis table of our multilevel model, based on exploratory data. The outcome variable is the logit of the clickthrough percentage on an article based on its headline. There is a significant negative interaction between headline concreteness and mean test concreteness, implying that the effect of headline concreteness on clickthrough rates varies negatively with mean test concreteness.

	Dependent variable:
	Logit(Clickthrough percentage)
Fixed Effects	
Headline Concreteness	0.274^{**}
	(0.089, 0.459)
Mean Test Concreteness	-0.017
	(-0.120, 0.086)
Headline Concreteness × Mean Test Concreteness	-0.089^{**}
	(-0.149, -0.029)
Constant	-4.457^{***}
	(-4.774, -4.139)
Random Effects	
Variance (Headline Concreteness)	0.1212
	($SD = 0.3481$)
Variance (Intercept)	0.4895
	($SD = 0.6996$)
Observations(i)	9,101
Observations(j)	2,335
Log Likelihood	$-41{,}902.450$
Akaike Inf. Crit.	83,818.900
Bayesian Inf. Crit.	83,868.710
Note:	* p<0.05; ** p<0.01; *** p<0.001

We also test the second and third hypotheses that the relationship between mean concreteness and headline concreteness might explain why prior experiments have observed both positive and negative effects. As a first step, we visualize the conditional effects of the interaction for different values of mean test concreteness. Supplementary Figure 2 illustrates that the slope is positive for lower values of mean test concreteness and negative for higher values of mean test concreteness. This intuitively implies that, for the pilot dataset, clickthrough rates vary positively with headline concreteness when all headlines

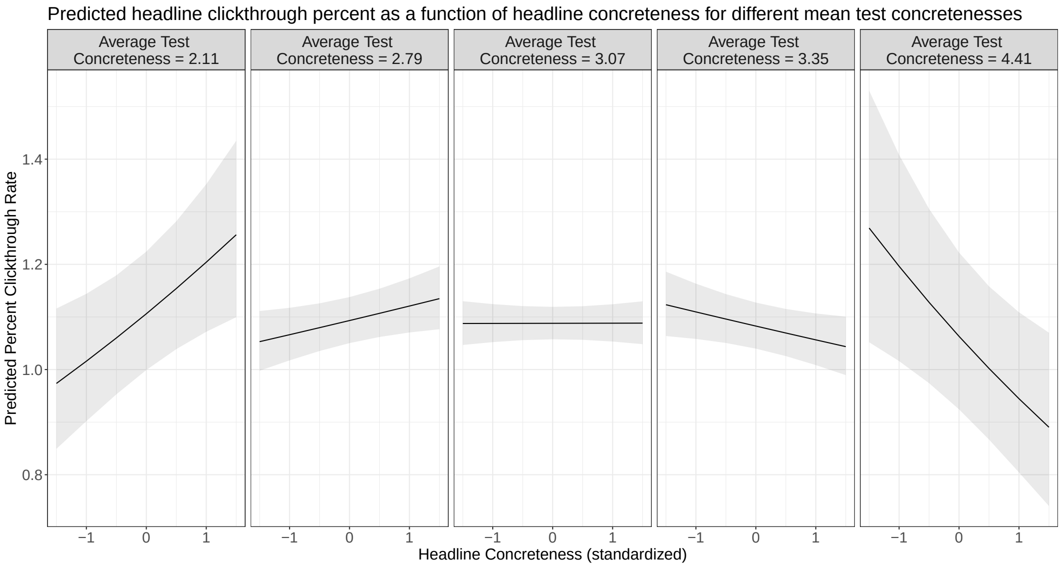

Supplementary Figure 2. Estimated marginal means of headline concreteness on predicted percent clickthrough rate, for different levels of mean test concreteness. The levels of mean test concreteness selected are: lowest value (2.11, left), one standard deviation below the mean (2.79, second from left), mean (3.07, middle), one standard deviation above the mean (3.35, second from right), highest value (4.41, right). The headline concreteness is depicted from -1 (2 standard deviations below the mean) to 1 (2 standard deviations above the mean). Overall, we observe an upside-down U-shape, where clickthrough rates appear to vary positively with headline concreteness when average test concreteness is lower, vary negatively with headline concreteness when average test concreteness is higher, and not vary when average test concreteness is in the middle.

in a test are low, and clickthrough rates vary negatively with headline concreteness when all headlines in a test are high. To confirm this intuition statistically, we perform simple slopes tests, which allow us to calculate the expected slope and its confidence interval for the interaction between headline concreteness and mean test concreteness at specific values of headline concreteness. We use the *reghelper* R package to test the simple slopes, and an online calculator[1] to calculate the bounds of mean test concreteness for which the slope is significant and visualize the analysis results. At the lower bound, the slope of the interaction is positive and significant for mean test concreteness values less than 2.82 (26.2% of tests in the pilot dataset), and the slope is negative and significant for mean test concreteness values greater than 3.33 (23.8% of tests in the pilot dataset). If an A/B test consists of two headlines that differ by one unit of standardized concreteness and which collectively have a low mean concreteness of 2.11, our model estimates that the clickthrough rate of the higher-concreteness headline will be 0.085 higher on the log-odds scale than the low-concreteness headline, and thus be more likely to be clicked on. Conversely, if an A/B test consists of two headlines that differ by one unit of concreteness, with a high mean concreteness of 4.41, our model estimates that the higher-concreteness headline will have a clickthrough rate that is 0.119 lower on the log odds scale than the than the low-concreteness headline, and thus be less likely to be clicked on. These relationships are illustrated by Supplementary Figure 3. After Holm's adjustment, the p-value for both H2 and H3 is adjusted to < 0.011. Since there is a significant lower bound and upper bound for the slope of the interaction within the range of possible mean test concreteness values, we reject the null hypothesis of no relationship for H2 and H3 in this exploratory pilot data. If this pilot analysis were our final analysis, we

[1] http://www.quantpsy.org/interact/hlm2.htm

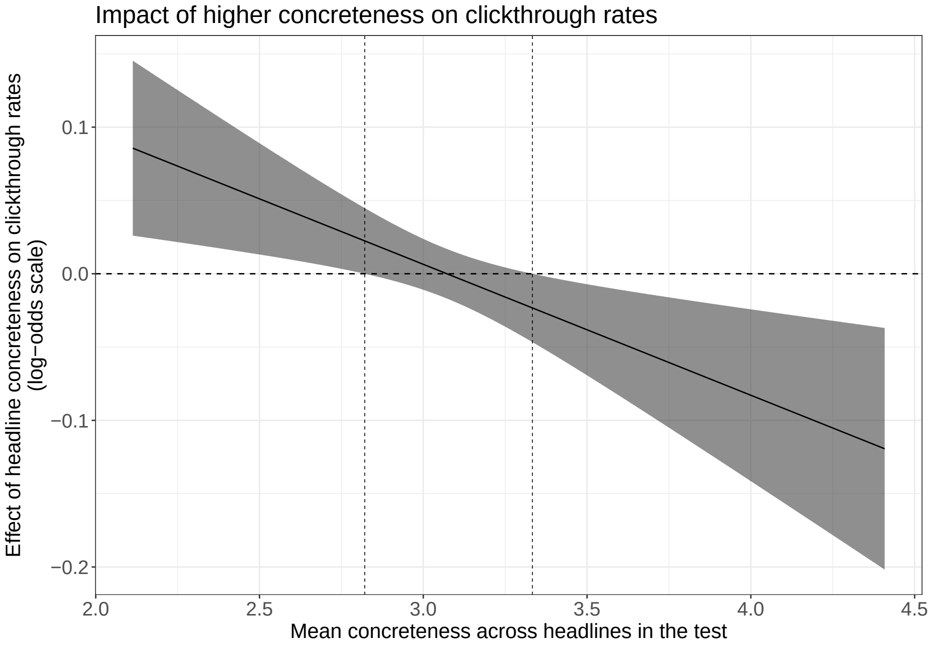

Supplementary Figure 3. Example illustration of the Johnson-Neyman technique based on exploratory data: Figure shows the estimated effect (y-values) of headline concreteness on the clickthrough percent as a function of mean test concreteness (x-values). Because the confidence intervals cross zero (marked by vertical lines at 2.82 and 3.33), the figure implies that more concrete headlines correlate significantly with higher clickthrough rates at lower levels of concreteness and lower clickthrough rates at higher levels of concreteness.

would conclude that at lower average test headline concreteness, higher headline concreteness would have a positive effect, but that at higher average test headline concreteness, higher headline concreteness would have a negative effect. This trend suggests there is an "optimal" level of concreteness, where the predicted clickthrough rate as a function of concreteness is maximised.

4 Randomization Problems in the Upworthy Research Archive

Since sending in this manuscript for review, further analyses of the Upworthy dataset have revealed that there was treatment imbalance for some of the experiments which occurred between June 25th, 2013 and January 10, 2014. Based on statistical analysis and interviews, the Upworthy Research Archive dataset has been updated to label roughly 22% of experiments as not having verifiable randomization. The dataset creators hypothesize that this randomization imbalance resulted from a third-party web cache that was showing repeated copies of the same experiment arm to participants rather than randomly assigning them. For that reason, the authors recommend omitting experiments between those dates from any causal analysis. The rest of the experiments in the archive are unaffected by this issue (1).

To address this issue, we have excluded all experiments in the affected period from the confirmatory analysis reported in the main paper.

5 Full Analysis Inclusive Of Unreliable Experiments in the Upworthy Research Archive

Because our final analysis considers a subset of experiments of those we planned to analyze, we also report the full analysis that we proposed in the Stage 1 registered report—inclusive of experiments with unreliable randomization. We find that the results and conclusions are not materially different when these unreliable experiments are included or excluded from the analysis.

Using the full dataset, we applied the headline concreteness metric to the 22,743 tests and 105,551 headlines that are part of the confirmatory data set. 87,011 of the pilot headlines can be tagged using our headline concreteness metric, so we continued the analysis with 82.4% of headlines included. When sub-selecting valid tests for comparison, we identify 11,158 one-image tests that meet our definition of a valid test, which include 43,601 non-unique headlines (37,614 unique headlines) tagged with concreteness.

We summarize the results of the binomial, random-intercepts and random-slopes multilevel model with an interaction between individual-level *headline concreteness* and group-level *test concreteness average* in Table 2.

Supplementary Table 2. Analysis table of the multilevel model. The outcome variable is the logit of the clickthrough percentage on an article based on its headline. There is a significant negative interaction between headline concreteness and mean test concreteness, implying that the effect of headline concreteness on clickthrough rates varies negatively with mean test concreteness.

	Dependent variable:
	ctr
concreteness_centered_standardized	0.134^{**}
	(0.045, 0.222)
test_concreteness_avg	0.034
	(−0.014, 0.081)
concreteness_centered_standardized:test_concreteness_avg	-0.047^{**}
	(−0.075, −0.018)
Constant	-4.592^{***}
	(−4.740, −4.444)
Observations (i)	43,601
Observations (j)	11,158
Log Likelihood	−201,380.000
Akaike Inf. Crit.	402,774.000
Bayesian Inf. Crit.	402,834.800
Note:	* $p < 0.05$; ** $p < 0.01$; *** $p < 0.001$

With the full dataset, the interaction between headline concreteness and average concreteness is negative at -0.047 ($p < 0.002$). The negative value of the interaction reflects that we expect the relationship between concreteness and clickthrough rate to be less for higher values of mean test concreteness. After performing the Holm correction, p is adjusted to < 0.003. Since the p-value is less than 0.05, and the coefficient is negative, we reject the null hypothesis of no relationship (H1). We thus

draw the same conclusion as in the full dataset.

We also tested the second and third hypotheses that the relationship between mean concreteness and headline concreteness might explain why prior experiments have observed both positive and negative effects. Once again, we perform simple slopes tests, which allow us to calculate the expected slope and its confidence interval for the interaction between headline concreteness and mean test concreteness at specific values of headline concreteness. At the lower bound, the slope of the interaction is positive and significant for mean test concreteness values less than 2.43 (3.8% of tests in the pilot dataset), and the slope is negative and significant for mean test concreteness values greater than 3.03 (53.8% of tests in the pilot dataset). If an A/B test consists of two headlines that differ by one unit of standardized concreteness and which collectively have a low mean concreteness of 2.03, our model estimates that the clickthrough rate of the higher-concreteness headline will be 0.04 higher ($holm - adjusted p < 0.015$) on the log-odds scale than the low-concreteness headline, and thus be more likely to be clicked on. Conversely, if an A/B test consists of two headlines that differ by one unit of concreteness, with a high mean concreteness of 4.41, our model estimates that the higher-concreteness headline will have a clickthrough rate that is 0.07 lower ($holm - adjusted p < 0.0001$) on the log odds scale than the than the low-concreteness headline, and thus be less likely to be clicked on. Thus, including the full dataset also confirms H2 and H3, as in the main document.

6 Lists of Headlines

These headlines would be considered too vague:

- *The Most Inspiring And Heartfelt Reason To Make Hilariously Inappropriate Jokes Ever*, Concreteness: 2.01
- *It Never Occurred To This First That She Would Be A First Anything*, Concreteness: 2.13
- *"The Special Effects Are Stunning, But The Real Event That Inspired It Is Truly Astonishing"*, Concreteness: 2.18
- *"What's Better Than A Celebrity Talking About Something Important? A Smart Celebrity Doing It."*, Concreteness: 2.42
- *"Why You Might Risk Your Life For Someone You've Never Even Met"*, Concreteness: 2.47

These headlines would be considered too concrete:

- *"An Anti-Gay Scoutmaster Gets A Reality Lesson From A Straight Eagle Scout And A Gay News Anchor"*, Concreteness: 3.26
- *"What One Big Oil Company Doesn't Want To Tell You About That Big Pipeline They Want To Build"*, Concreteness: 3.48
- *"Here's All The 'Scary Stuff' He Found When He Looked At Hundreds Of Studies On Fluoride In Our Water"*, Concreteness: 3.50

- *”He Was About To Take His Own Life — Until A Man Stopped Him. Here He Meets Him Face To Face Again.”*, Concreteness: 3.54
- *In 1920, He Took On Wall Street, Which Put Him In Jail. Then He Got A Million Votes For President.*, Concreteness: 3.64

References

Matias, J. N., Munger, K., Aubin Le Quere, M. & Ebersole, C. The Upworthy Research Archive, a time series of 32,487 experiments in U.S. media. *Sci. Data* **8**, 195, doi:10.1038/s41597-021-00934-7 (2021).
